# Role of Pericytes in Cardiomyopathy-Associated Myocardial Infarction Revealed by Multiple Single-Cell Sequencing Analysis

**DOI:** 10.3390/biomedicines11112896

**Published:** 2023-10-26

**Authors:** Yanqiao Lu, Huanhuan Huo, Feng Liang, Jieyuan Xue, Liang Fang, Yutong Miao, Lan Shen, Ben He

**Affiliations:** Department of Cardiology, Shanghai Chest Hospital, School of Medicine, Shanghai Jiao Tong University, Shanghai 200030, China; luyanqiao2013@live.com (Y.L.); huohuanhuan540@126.com (H.H.); liangfengwind@sjtu.edu.cn (F.L.); jieyuanxue@outlook.com (J.X.); fangliang0@foxmail.com (L.F.); miaoyutong8128@163.com (Y.M.)

**Keywords:** cardiomyopathy, myocardial infarction, pericytes, *SMAD3*

## Abstract

Acute myocardial infarction (AMI) is one of the leading causes of cardiovascular death worldwide. AMI with cardiomyopathy is accompanied by a poor long-term prognosis. However, limited studies have focused on the mechanism of cardiomyopathy associated with AMI. Pericytes are important to the microvascular function in the heart, yet little attention has been paid to their function in myocardial infarction until now. In this study, we integrated single-cell data from individuals with cardiomyopathy and myocardial infarction (MI) GWAS data to reveal the potential function of pericytes in cardiomyopathy-associated MI. We found that pericytes were concentrated in the left atrium and left ventricle tissues. *DLC1*/*GUCY1A2*/*EGFLAM* were the top three uniquely expressed genes in pericytes (*p* < 0.05). The marker genes of pericytes were enriched in renin secretion, vascular smooth muscle contraction, gap junction, purine metabolism, and diabetic cardiomyopathy pathways (*p* < 0.05). Among these pathways, the renin secretion and purine metabolism pathways were also found in the process of MI. In cardiomyopathy patients, the biosynthesis of collagen, modulating enzymes, and collagen formation were uniquely negatively regulated in pericytes compared to other cell types (*p* < 0.05). *COL4A2*/*COL4A1*/*SMAD3* were the hub genes in pericyte function involved in cardiomyopathy and AMI. In conclusion, this study provides new evidence about the importance of pericytes in the pathogenesis of cardiomyopathy-associated MI. *DLC1*/*GUCY1A2*/*EGFLAM* were highly expressed in pericytes. The hub genes *COL4A2*/*COL4A1*/*SMAD3* may be potential research targets for cardiomyopathy-associated MI.

## 1. Introduction

Cardiovascular disease (CVD) is one of the leading causes of death worldwide, accounting for over 4 million deaths in Europe each year [1]. Acute myocardial infarction (AMI) is a life-threatening type of CVD that can cause malignant arrhythmia and sudden cardiac death [2]. When an artery becomes acutely blocked, blood flow is disrupted and the heart is unable to supply blood normally. Severe myocardial ischemia and hypoxia result in the damage and death of myocardial cells [3]. After the acute phase, adverse ventricular remodeling reduces cardiac function, resulting in heart failure and increased mortality [4]. In developed countries, AMI is one of the leading causes of death [5]. Despite rapid advances in therapeutic technology and out-of-hospital management [6], morbidity and mortality rates remain around 5% [7]. AMI with cardiomyopathy is a specific type of AMI. It is reported that patients with AMI and cardiomyopathy experience poorer long-term outcomes [8]. Few studies focus on the pathogenesis of AMI with cardiomyopathy. Most pathogenesis studies focus on the whole heart or major cell types in the heart, such as cardiomyocytes and macrophages [9,10,11]. The role of other cells in the pathogenesis of AMI with cardiomyopathy has not been well studied. 

Microvascular pericytes and the recently discovered adventitial pericyte-like progenitors are closely associated with small and large vessels, respectively [12]. The role of pericytes in cardiovascular diseases has been increasingly recognized. Firstly, pericytes are a safe cell type. Furthermore, vascular pericytes are more abundant than other types of cells in the heart [13]. Pericyte dysfunction, on the other hand, may be involved in the pathogenesis of CVD [14], such as the processes involved in the regulation of cardiac homeostasis, angiogenesis, blood flow regulation, coagulation process regulation, and so on. Pericytes have received increasing attention for their applications in cardiovascular diseases, due to their pleiotropic and powerful angiogenic capacities [15]. The multipotent pericytes in capillaries are EphA7-positive, with angiogenic abilities [16]. During the progress of AMI, cardiac pericytes can migrate to the infarction zone and engage in the remodeling of the injured heart [17]. Cardiac pericytes upregulate the fibrosis-associated genes and matrix synthetic profiles, which promote the maturation of infarct vasculature in AMI [18]. Thus, understanding the crucial roles of pericytes in AMI may be helpful in identifying potential therapy targets for AMI.

**Figure 1:**
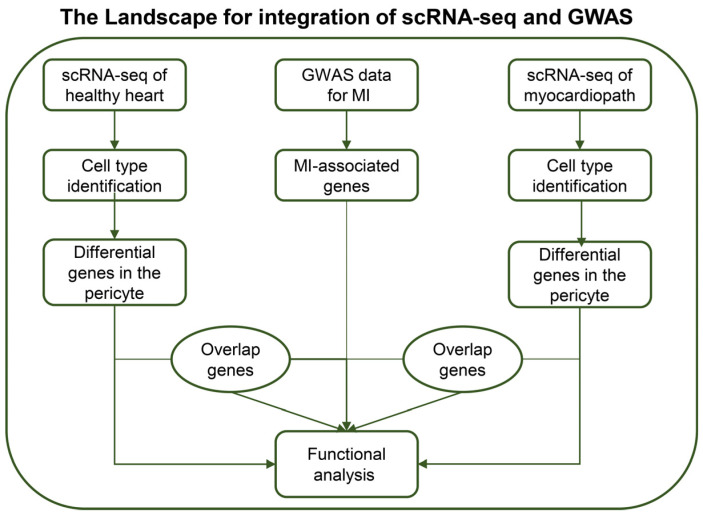
Introduction of the datasets and framework of analysis in this study.

In this study, we integrated single-cell RNA-seq data and MI GWAS data to reveal the mechanisms by which differential cell types influence myocardial infarction (MI) processing. Briefly, through the functional analysis and overlapping genes, integrating GWAS and single RNA-seq data, the function of pericyte may differ between cardiomyopathy and healthy individuals associated with MI (the workflow is shown in Figure 1).

## 2. Materials and Methods

### 2.1. Dataset Download

In this study, gene counts of single cells in the absence of overt cardiac disease were downloaded from the Broad Institute’s Single Cell Portal (SCP498, accessed on August 2020) [19]. GWAS data for MI (myocardial infarction) were obtained from the GWAS Catalog GCST011365 [20]; another dataset of single cells from cardiomyopathy patients was accessed through the Broad Institute’s Single Cell Portal (https://singlecell.broadinstitute.org/single_cell) under project ID SCP1303 (accessed on February 2021) [21]. There were 16 hearts from normal patients, 15 hearts from hypertrophic cardiomyopathy (HCM) patients, and 11 hearts from dilated cardiomyopathy (DCM) patients included in the following analysis.

### 2.2. Single-Cell RNA-Seq Analysis

The common processes of filtering, differential gene screening, dimensionality reduction, and clustering were carried out using the single-cell RNA-Seq analysis R package, Seurat (v.2.3.0) [22], according to a previous study. We screened cells in both single-cell experiments for those with fewer than 200 genes per 1000 UMIs to weed out low-quality cells, and eliminated cells with more than 5800 UMIs to weed out doublets that were more likely to exist. We used the Bioconductor developed by Lun et al. and implemented it in the R packages Scran (v.1.6.2) [23] and Scater (v.1.0.3) [24]. Principal component analysis was used to dimensionally reduce the expression matrix. The FindCluster function in Seurat used a graph-based clustering approach to identify clusters. In order to find rare populations, we used modularity-based clustering, a sensitive technique that occasionally over-partitions larger clusters. We used the model-based analysis of Single-cell Transcriptomics test for all single-cell differential expression tests.

### 2.3. Fine-Mapping

The causal variants were identified in the GWAS data. *p* < 5 × 10^−8^ was used as a filter to obtain loci with genome-wide significance. We used statistical fine-mapping across thousands of trait-associated loci, using a single evidence score to resolve association signals and connect each variant to its proximal and distal target genes. The Odds Ratio was used to evaluate the effect size of the MI GWAS data. 

### 2.4. Pathway Enrichment Analysis

gProfiler (http://www.biit.cs.ut.ee/gprofiler/, accessed on 2007) was used for the pathway enrichment analysis. The Benjamini–Hochberg FDR was used to determine the significance threshold and 0.05 was used as a threshold in gProfiler. Gene Ontology (http://www.geneontology.org/, accessed on 2000), molecular pathways of Reactome (http://www.reactome.org/, accessed on 2005), and KEGG (https://www.genome.jp/kegg/, accessed on 1995) were applied to perform bioinformatics analysis.

### 2.5. Statistics

The differentially expressed genes were analyzed using the R package “limma” and defined as *p* < 0.05 and |log (FC)| > 1. All results are shown as mean SEM. For comparisons between the two groups, a two-tailed, unpaired Student’s *t*-test was used. In the present study, no randomization was used. *p* < 0.05 was used to define significant differences. Prism (version 9, San Diego, CA, USA) and R (version 4.1.2, New Zealand) software were used for the statistical analysis.

## 3. Results

### 3.1. Identifying Unique Genes and Function Pathways of Pericytes in Healthy Donors

**Figure 2:**
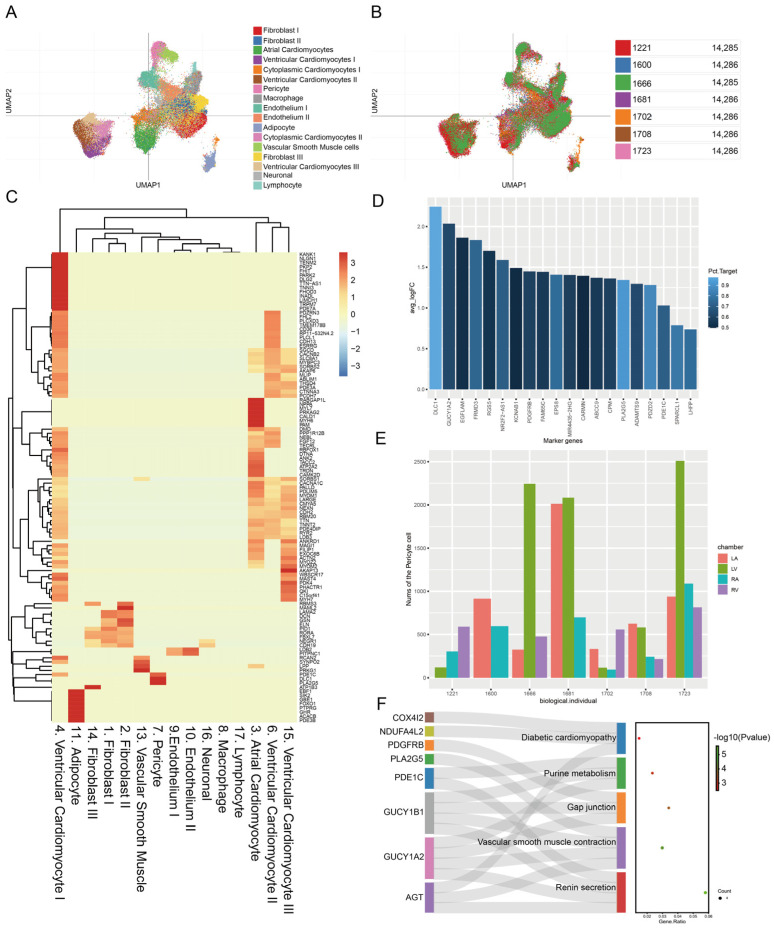
The single-cell landscape of healthy donors. (**A**) The cluster of the cell types in the hearts of healthy donors. (**B**) The cluster of the different individuals in the hearts of healthy donors. (**C**) The unique marker of the different cell types. (**D**) The average fold change of the marker gene in pericytes. (**E**) The number of pericytes across different chambers and individuals. (**F**) The functional enrichment for pericytes’ marker genes. The greener the spot is, the more significant the statistical difference is (*p* < 0.05). LV, left ventricle; LA, left atrium; RA, right atrium; RV, right ventricle.

To understand the proportion of different cell types in the heart, we used the scRNA-seq dataset of healthy donors to identify cell types (SCP498) [19]. A total of 17 distinct cell clusters were identified after unsupervised Louvain clustering at a resolution of 1.0. As present in Figure 2A,B, we showed cell cluster distributions using individual-specific uniform manifold approximation and projection representations. Then, we further analyzed the differential genes between these cells for each cell type, as shown in Figure 2C. We found that *DLC1*/*GUCY1A2*/*EGFLAM* were the top three uniquely expressed genes in the pericytes (Figure 2D). Also, we found that pericytes were concentrated in the left atrium and left ventricle tissues across several individuals (Figure 2E). Interestingly, we found that pericytes were involved in renin secretion, vascular smooth muscle contraction, gap junction, purine metabolism, and diabetic cardiomyopathy, according to the functional analysis (*p* < 0.05, Figure 2F).

### 3.2. The Landscape of MI-Associated Genes

**Figure 3:**
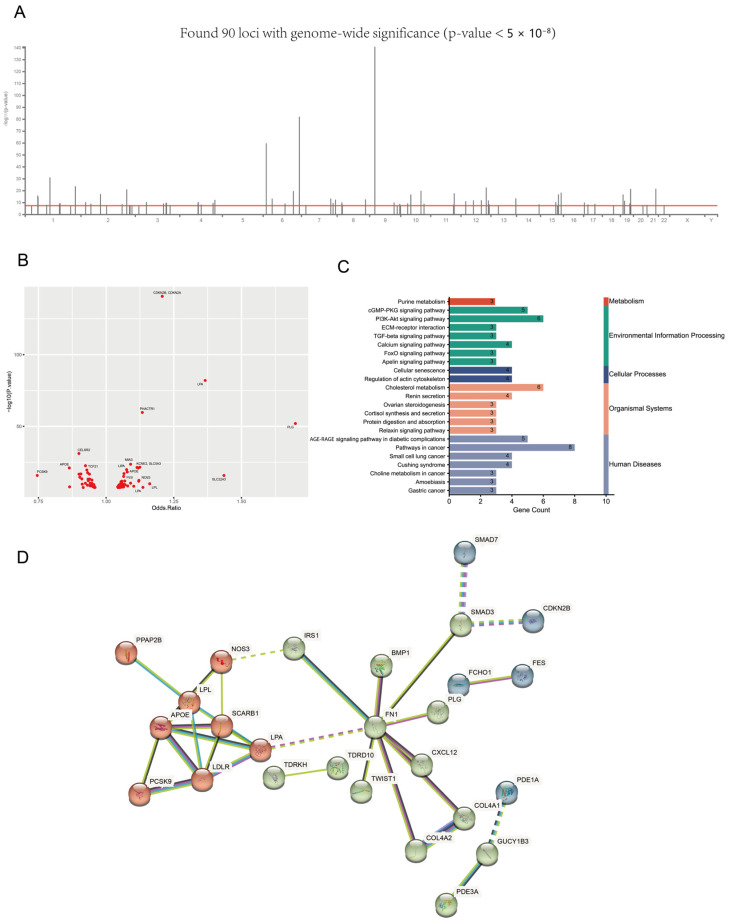
The MI-associated genes identified by the GWAS. (**A**) The distribution of the significant loci. (*p* < 5 × 10^−8^) (**B**) The genes associated with the loci; the larger the value on the Y-axis is, the more significant the statistical difference is. (*p* < 0.05). (**C**) The KEGG enrichment for the MI-associated genes. (**D**) The PPI network for the MI-associated genes.

Although the mortality of MI is decreasing due to developments in medicine, MI still imposes a substantial disease burden on patients. How to improve the prevention and treatment of AMI remains a problem to be solved. The GWAS data of MI patients can help us understand the pathogenesis of MI. Through the GWAS data [20], we applied a fine-mapping technique to identify causal loci and then defined the nearest genes with each causal locus as MI-associated genes. Overall, we found 90 independent loci and 87 MI-associated genes (*p* < 0.05, Figure 3A,B). Then, we identified the pathways involved in these genes (Figure 3C). Among them, we found that the renin secretion and purine metabolism pathway were also found in the pathways of pericytes from healthy donors, which indicates the potential involvement of pericyte in the MI process. Furthermore, we found that MI-associated genes had a strong preference in the PPI (protein–protein interaction) network, according to the STRING database (Figure 3D). However, we did not find any overlap genes between the differential genes in healthy pericytes and MI-associated genes. This result reminds us that the dysfunction of pericytes may be involved in the MI process.

### 3.3. Identifying Function Pathways of Pericytes in Cardiomyopathy Patients

**Figure 4:**
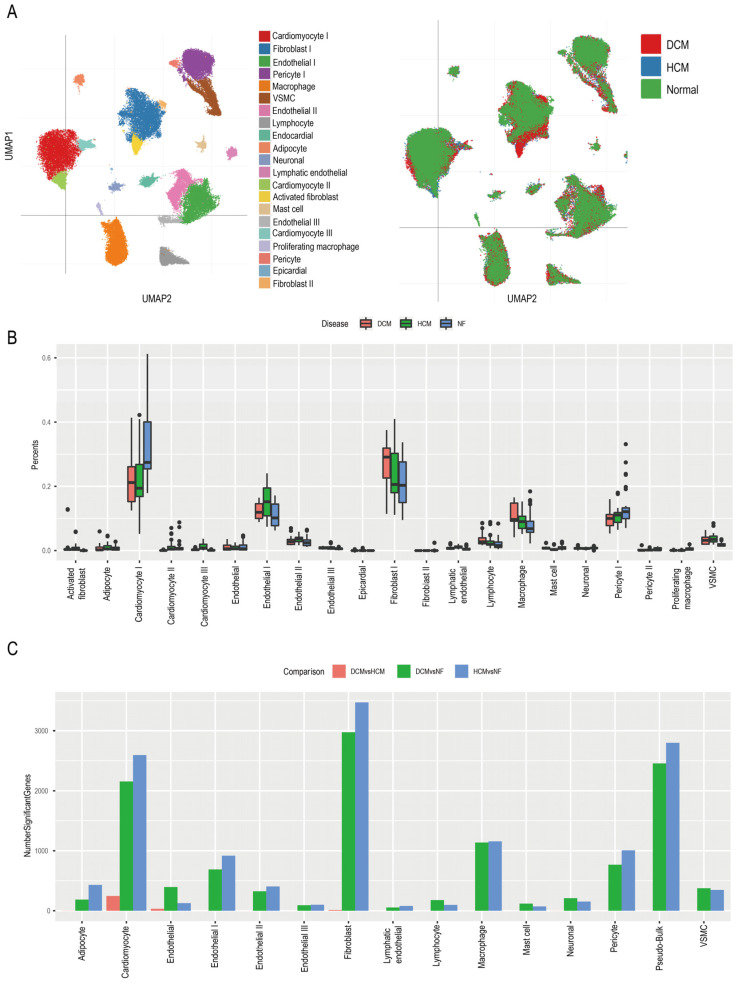
The landscape of single-cell RNAseq of cardiomyopathy patients. (**A**) The cluster of the cell subsets in the hearts from DCM, HCM, and non-heart failure patients. (**B**) Distribution of different cell types in the heart tissues of DCM, HCM, and non-heart failure patients. (**C**) The number of significant genes in different cell types of DCM, HCM, and non-heart failure patients. Significant genes were defined as *p* < 0.05 and |log (FC)| > 1.

**Figure 5:**
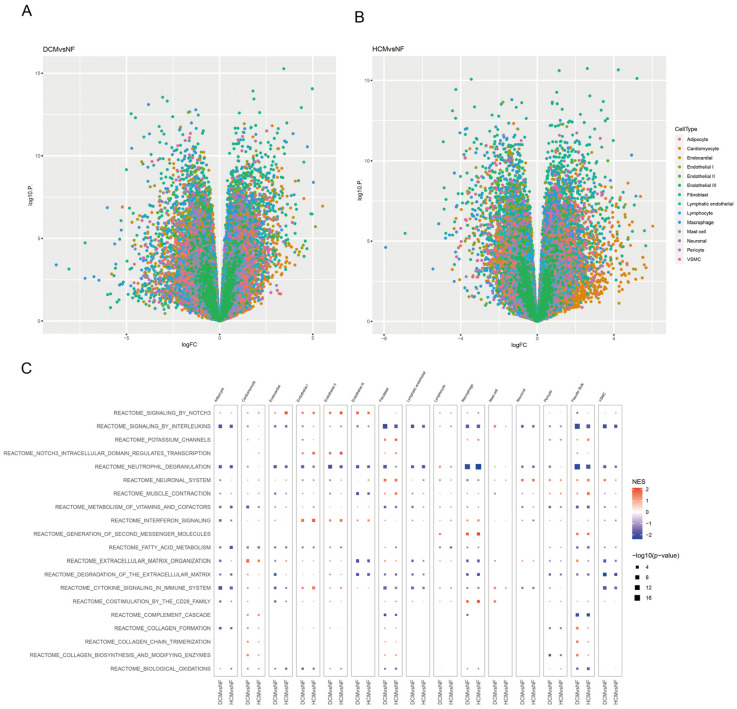
The function of the significant genes of cardiomyopathy patients in different cell types. (**A**) The volcano plot of the differential genes in different cell types between DCM and non-heart failure. Differential genes were defined as *p* < 0.05 and |log (FC)| > 1. (**B**) The volcano plot of the differential genes in different cell types between HCM and non-heart failure. Differential genes were defined as *p* < 0.05 and |log (FC)| > 1. (**C**) The gene enrichment for different cell types in different comparisons. The bigger the block, the more significant the statistical difference. (*p* < 0.05).

Next, we tried to figure out the relationship between cardiomyopathy-associated MI and pericyte function. Thus, we used the single-cell RNA-seq of cardiomyopathy patients, a group associated with a potential cause of the poor long-term prognosis of MI [8,25], to identify the functional transformations in pericytes. Both HCM and DCM are the most common clinically observed cardiomyopathies. The physiopathology mechanisms of these two kinds of cardiomyopathies are not identical. Therefore, we analyzed the single-cell datasets from both HCM and DCM patients to reveal the function of pericytes in each (SCP1303) [21]. Firstly, we found 20 cell types across these datasets (Figure 4A). Then, comparing the percentages of different cell types among different disease groups, we found that Cardiomyocyte I, Fibroblast I, Endothelial I, Macrophage, and Pericyte I were the top five cell types in each disease group (Figure 4B). Cardiomyocyte I, Fibroblast I, Endothelial I, Macrophage, and Pericyte I were also the top six cell types in the number of significant genes (Figure 4C and Figure 5A,B). According to the significant genes in each of these cell types, we conducted pathway enrichment analyses and compared them to those of normal patients. We found that signaling by interleukins, neutrophil degranulation, fatty acid metabolism, degradation of the extracellular matrix, and Y-Toxin signaling in the immune system were majorly negatively regulated in all cell types, and interestingly, biosynthesis of collagen, modulating enzymes, and collagen formation were uniquely negatively regulated in the pericytes (*p* < 0.05, Figure 5C). These results indicate that biosynthesis of collagen, modulating enzymes, and collagen formation may be the key pathways of pericytes in the pathophysiology of cardiac hypertrophy.

### 3.4. Hub Genes of Pericytes Involved in the MI Process

**Figure 6:**
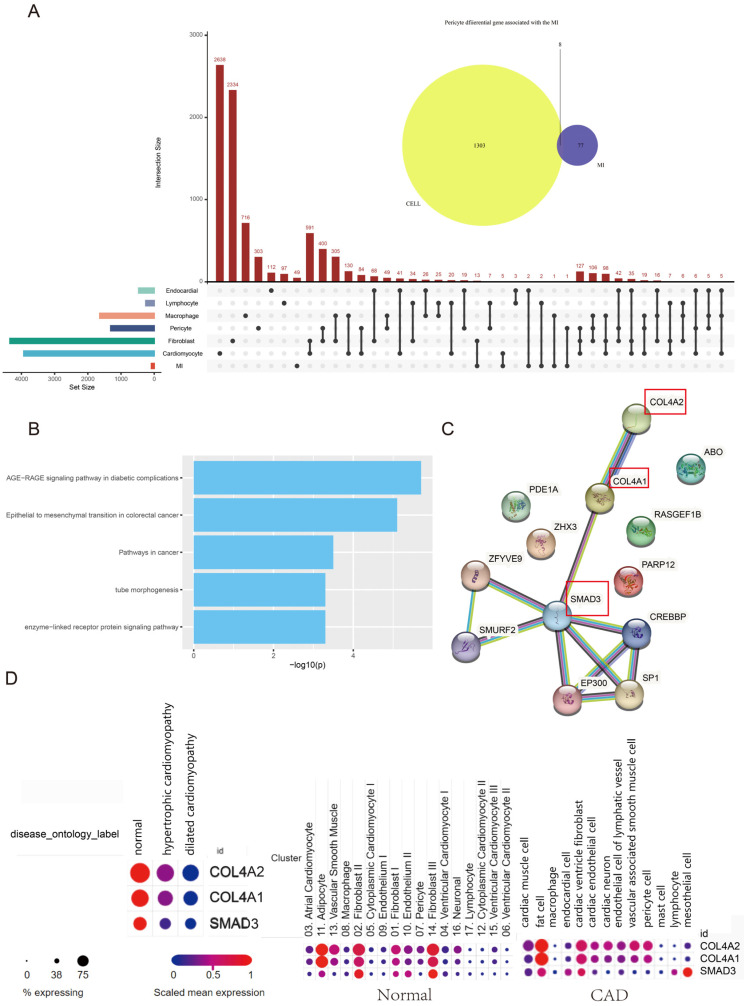
The integration of MI-associated genes and cardiomyopathy-associated genes. (**A**) The landscape of differential genes in different cell types overlapped with the MI-associated genes. (**B**) The gene enrichment for overlapping genes between pericytes and MI-associated genes. The larger the value on the X-axis, the more significant the statistical difference. (*p* < 0.05) (**C**) The hub genes for overlapping genes between pericytes and MI-associated genes. The red box showed the hub genes involved in the MI process. (**D**) The expression profile of hub genes across different disease statuses.

To further explore pericytes’ function during the cardiomyopathy-associated MI process, we combined the MI GWAS dataset and single-cell RNA seq data from cardiomyopathy for analysis. We used differential genes to obtain cardiomyopathy-associated genes that overlapped with MI-associated genes (Figure 6A) in the top five cell types. We found that this overlap in genes was observed in Cardiomyocyte, Fibroblast, Pericyte, Macrophage, Lymphocyte, and Endocardial, varying from 1 to 13. Among them, the number of overlapping genes between the pericytes of cardiomyopathy and MI-associated genes was eight (Figure 6A). According to the functional analysis, enzyme-linked receptor protein signaling pathways, tube morphogenesis, pathways in cancer, epithelial to mesenchymal transition in colorectal cancer, and AGE−RAGE signaling pathways in diabetic complications were identified by these eight genes (*p* < 0.05, Figure 6B). PPI analysis found that the *COL4A2*/*COL4A1*/*SMAD3* genes were the hub genes of the pericytes involved in the MI process (Figure 6C). We found that the *COL4A2*/*COL4A1*/*SMAD3* were all expressed at lower levels in HCM and DCM patients but higher in pericytes (Figure 6D). These results may indicate that the regulation of the *COL4A2*/*COL4A1*/*SMAD3* pathway in pericytes may be a potential target that will affect the process of cardiomyopathy-associated MI.

## 4. Discussion

Since their establishment in 2009, single-cell technologies have grown in popularity as their scale and cost have decreased significantly [26]. Single-cell techniques can be employed to answer a variety of questions, from determining the relationship between cell types and CVD to determining how gene expressions and regulations change in disease [27,28,29,30]. Several studies have used single-cell techniques to investigate AMI pathogenesis [29,31]. Jun Qian et al. discovered 27 cell clusters in 82,550 AMI patients’ peripheral blood cells, including monocytes and T/B/NK cells [29]. Jun Qian et al. reported that *CCL5*, *TLR7*, and *CX3CR1* were significantly higher in patients with plaque rupture [29]. In addition, Song et al. defined a total of seven cell clusters marked by marker genes into five cell types and identified five hub genes involved in AMI progression (*ATM*, *CARM1*, *CASP8*, *CASP3*, and *PPARG*) using the scRNA-seq method [32]. In this study, we integrated single-cell RNA-seq data with MI GWAS data to reveal the mechanisms through which differentiated cell types influence cardiomyopathy-associated MI processing. We used scRNA-seq datasets from healthy donors to identify different cell types and their proportions in healthy hearts. In our research, a total of 17 distinct cell clusters were observed at a resolution of 1.0 after unsupervised Louvain clustering.

Microvascular pericytes are the cells that surround endothelial cells in capillaries and microvessels in most organs. The function of cardiac microvessels is important in keeping heart function and improving post-MI recovery. Cardiac-derived pericytes may be more prone to involvement in the function of cardiac microvessels. The gene heterogeneity among pericytes is very complicated, and in recent years, studies about pericyte function have been increasing. Single-cell analysis has revealed that pericytes make up a large proportion of the heart [13], implying that they play a vital role in the heart. It has been reported that cardiac pericytes can promote vascular homeostasis and attenuate adverse cardiac remodeling after AMI [17,18]. Additionally, the critical role of pericytes in the regulation of blood flow is widely recognized [33]. Augustin et al. found that the lack of pericyte-expressed Tie2 might lead to pro-migratory phenotype [34]. Moreover, it has been reported that the Ca^2+^ concentration of pericytes regulates TMEM16A and reduces post-ischemia blood flow [35]. However, studies on the relationship between pericytes and heart function are still limited until now.

A recent study from Teichmann et al. showed that pericytes account for a large proportion of the heart [13]. In this study, we found that pericytes from several individuals were mainly present in the left atrium and left ventricle tissues. In addition, *DLC1*/*GUCY1A2*/*EGFLAM* were found to be the top three uniquely expressed genes in pericytes. Zhang et al. utilized bioinformatics analysis to screen for biomarkers related to MI and revealed that *DLC1* is the most important node in MI [36]. So far, there is still a lack of study on the role of *DLC1* in pericytes. *EGFLAM* is found in a variety of organs and tissues, including the brain, endocrine tissue, and muscle tissue [37]. *EGFLAM* has been linked to defects in photoreceptor synapse function in congenital muscular dystrophies, such as muscle–eye–brain disorders caused by dystrophic glycosylation [38]. It has also been reported that *EGFLAM* can be a risk factor for dissecting aortic aneurysms [38]. An exome-wide association study identified *EGFLAM*, *SPATC1L*, and *RNASE13* as novel susceptibility loci for aortic aneurysms in the Japanese population [38]. In our research, we also found that pericytes in normal hearts are involved in renin secretion, vascular smooth muscle contraction, gap junctions, purine metabolism, and diabetic cardiomyopathy, according to functional analyses. It has been reported that ACE2 is highly expressed in heart pericytes, inducing microvascular dysfunction [39]. This study suggests that the renin–angiotensin system and related genes are important to pericyte function. We found that renin secretion and purine metabolism are also present in the MI pathway through the GWAS, suggesting that pericytes have the potential to participate in the MI process. 

Cardiomyopathy complicated by MI is relevant to a poor prognosis. Both cardiomyopathy and MI can evolve into heart failure. To study the pathogenesis of cardiomyopathy-associated MI in depth, we compared the percentages of different cell types in the different disease groups (HCM and DCM) in the database [21]. Cardiomyocyte I, Fibroblast I, Endothelial I, Macrophage, and Pericyte I were the top five cell types in each disease group. Signaling by interleukins, neutrophil degranulation, fatty acid metabolism, degradation of the extracellular Matrix, and Y-toxin signaling in the immune system were uniquely negatively regulated in all cell types. The extracellular matrix consists of collagen, elastin, fibronectin, and other proteoglycans, which are vital for damage repair and signal transduction. It was reported that collagen plays a special role in maintaining the integrity of blood vessels and in the processes of thrombosis and scarring [40]. Collagen fibers, in addition to providing tensile strength and stiffness to the heart muscle, also serve as structural scaffolding for muscle cells. According to several studies, coronary occlusion not only kills muscle cells but also destroys collagen [40]. Thus, collagen damage impairs muscle cell support, reduces myocardial strength and stiffness, and allows infarcted tissue to expand. The extracellular matrix metalloproteinases degrade collagen, which is normally dormant in the myocardium but is activated by ischemia [41]. The number of damaged collagen fibers in the rat heart increased over time after coronary occlusion, within the first four days after infarction, as measured by light microscopy [42]. All these results indicate that collagen may be meaningful to cardiomyopathy-complicated MI. Next, we used differential genes from cardiomyopathy patients to obtain genes that overlapped with MI-associated genes in the top five cell types. Eight genes were identified and several pathways were discovered through functional analysis. It seems that the EMT could improve the prognosis of MI patients. According to recent research, EMT is involved in the process of cardiac regeneration and repair [43,44]. Hence, changes in EMT-related genes might explain the poor prognosis of cardiomyopathy-associated MI. 

Our further PPI analysis found that the *COL4A2/COL4A1*/*SMAD3* genes are the central genes of pericytes involved in the MI process. We found that the expression of *COL4A2*/*COL4A1*/*SMAD3* was lower in both HCM and DCM, but higher in pericytes, which may suggest that pericytes can prevent the progression of MI by upregulating *COL4A2*/*COL4A1*/*SMAD3*. *COL4A1* and *COL4A2* encode the first and second chains of type IV collagen [45,46]. Type IV collagen is a critical component of basement membrane integrity and functionality [47]. Mutations in the *COL4A1*/*COL4A2* locus influence vascular cell survival, atherosclerotic plaque stability, and MI risk [48]. Apoptosis is induced by the silencing of *COL4A1* or *COL4A2* in vascular smooth muscle cells or endothelial cells. Similarly, shift mutations in *COL4A2* increase the rate of apoptosis in fibroblasts of mutation-carrying individuals [48]. So far, no study has proposed the effect of *COL4A1* or *COL4A2* on cardiomyopathy-associated MI. Smad2 and Smad3 signaling pathways located in cardiomyocytes and stromal cells are activated in infarcted myocardium [49]. Smad3 signal transduction enhances myofibroblast transdifferentiation and stimulates matrix preservation procedures [50]. In reperfusion infarct models, there is a total absence of Smad3 attenuating post-infarct remolding. The negative effects of Smad3 deletion include uncontrolled fibroblast proliferation and a misaligned myofibroblast array in the marginal region [51]. Smad3 signaling controls fibroblast activity and triggers integrin-mediated NOX2 expression. The infarcted heart is shielded from the effects of post-infarct dysfunction by the absence of cardiomyocyte-specific Smad3 [51,52]. Smad3 loss is related to reduced NOX2 levels, nitrosation stress, and MMP2 expression, which promotes the survival and growth of B cells, as well as the weakening of cardiomyocyte apoptosis in remodeled myocardium [51]. Additionally, Smad3-expressing macrophages guard the infarcted heart, promote phagocytosis, and control inflammation [53]. Additionally, Smad3 is crucial in the process of cardiomyopathy initiation and development. The expression of Smad2 and Smad3 aggravates myocardial fibrosis, and the abnormal regulation of the Smad pathway leads to more cardiac deaths [54]. Disturbing EphrinB2, which inhibits the TGF-β/Smad3 pathway in fibroblasts, can reduce fibrotic remodeling and improve heart function in cardiomyopathy models [55]. All these studies suggest that the *COL4A2*/*COL4A1*/*SMAD3* pathway may have a potential function in regulating the progress of cardiomyopathy-associated MI. However, how the *COL4A2*/*COL4A1*/*SMAD3* pathway regulates the process of cardiomyopathy-associated MI requires further study.

## 5. Conclusions

Overall, in this study, we integrated single-cell data from normal and CVD individual, along with MI GWAS data to reveal the function of pericytes in the cardiomyopathy-associated MI process. Using single-cell data from healthy donors, we found that pericytes were concentrated in the left atrium and left ventricle tissues across several individuals. Interestingly, we found that pericytes were involved in renin secretion, vascular smooth muscle contraction, gap junction, purine metabolism, and diabetic cardiomyopathy, according to the functional analysis. Next, MI-associated genes were identified through the GWAS data. Moreover, we found that the pericytes were the top five cell types in each disease group. Finally, we identified *COL4A2*/*COL4A1*/*SMAD3* as the hub gene in pericyte function involved in the cardiomyopathy-associated MI process. We believe this study provides new evidence proving that pericytes play a key role in the pathogenesis of cardiomyopathy-associated MI and could serve as potential therapy targets for the treatment of patients with cardiomyopathy-associated MI.

## 6. Limitations

However, the limitations of this study should not be overlooked. Firstly, this research was a secondary analysis of published single-cell sequencing databases and a GWAS database, which may cause certain deviations in the results. Secondly, the single-cell sequencing data of cardiomyopathy were not matched with the GWAS data of MI. It would be better to analyze data from patients with cardiomyopathy-associated MI directly, rather than combining two different datasets. Finally, the bioinformatics analysis only provided potential pathways involved in cardiomyopathy-associated MI. These results still need further verification through additional experiments.

## Data Availability

The datasets analyzed during the current study are available in the Broad Institute’s Single Cell Portal (SCP498, SCP1303) and GWAS Catalog GCST011365. All the original data are available from the corresponding author.

## Abbreviations

CVD	cardiovascular disease
AMI	acute myocardial infarction
MI	myocardial infarction
PPI	protein–protein interaction
HCM	hypertrophic cardiomyopathy
DCM	dilated cardiomyopathy
EMT	epithelial–mesenchymal transition
LV	left ventricle
LA	left atrium
RA	right atrium
RV	right ventricle
